# The Frequent Interdependence of Dislocated Kidney, Gall-Bladder Trouble and Appendicitis

**Published:** 1907-11

**Authors:** Earl Harlan

**Affiliations:** Cincinnati, O.


### "THE FREQUENT INTERDEPENDENCE OF DISLOCATED KIDNEY, GALL-BLADDER TROUBLE AND APPENDICITIS."*

By EARL HARLAN, M.D., Cincinnati, O.

A ten years' study of the subject has impressed the author with the serious import of "Dislocated Kidney."

He discusses the status of "Dislocated Kidney" as a pathological entity capable of producing symptoms of functional and physical derangement, primarily, within the organ itself, and, secondarily, on the part of the stomach, cholecyst, bowel and appendix.

He divides movable kidneys into two classes: First—Those with, and: Second—Those without clinical pathology, e. g. bedside symptoms, and calls those of the first division, "Movable Kidneys," in contradistinction to those of the second class which

---

(*Author's abstract of paper read before the Mississippi Medical Association, Columbus, Ohio. Oct. 6-10, 1907.)

he places in the category of "Dislocated Kidney," the latter term being more applicable as conveying pathological meaning.

From three to five per cent. of movable kidneys are of the second class, and therefore demand surgical interference.

He justifies the belief in the minds of those who doubt or scoff at the existence of the possible pathology of a movable kidney on the grounds that, although they may have seen many freely movable kidneys, they probably have not come into contact with a pathologically dislocated kidney, or if they have, they have been misled in the diagnosis of the latter. He supports the claim that a "dislocated kidney" may present the clinical picture of gastric irritation or ulcer, obstruction to the bile outflow with attacks of jaundice, partial obstruction of the intestine, or appendiceal irritation or appendicitis, without presenting any symptoms or conditions other than displacement, which would tend to involve the organ itself, pathologically speaking, with a number of reports of misoperated cases.

He cites the recent great work of Suckling of Birmingham, England, who proved that insanity is often produced by "dislocated kidney" and reports the cure of twenty-one cases who underwent the operation for correction of the pathological displacement. The diagnosis was made in these cases by measuring the amount of kidney excrement and comparing with that of the normal kidney. He also refers to the work of Harris of Chicago, in establishing the pathology of these cases.

He traces the relationship and pathological dependence of gastric trouble, bile-duct and bowel obstruction, and appendiceal irritation with colic with "dislocated kidney" and emphasizes the great importance of making an exact diagnosis in all cases of chronic indigestion with obscure symptoms and conditions, citing cases as stated, which had undergone a mistaken diagnosis and having been operated upon for appendicitis or gall-stones, wherein the primary lesion was a dislocated kidney.

He says that this lesion more often escapes notice and diagnosis than all other abdominal lesions combined.

He summarizes the salient features of his paper in the following paragraphs:

"First—That, as dependent pathology, accompanying the primary lesion of 'dislocated kidney'; Gall-bladder trouble, appendiceal irritation or appendicitis are of frequent concomitant occurrence.

"Second—That many cases have undergone operation for appendiceal trouble or gall-bladder trouble wherein the causative factor was a 'dislocated kidney,' the latter producing pressure interference to the bile outflow or bowel current.

"Third—That all cases presenting for abdominal treatment, complaining of indefinite and recurrent distress, discomfort and pain, these attacks being accompanied with the presence of an excess of stagnant gas at certain points in the bowel, and in which the involvement of the stomach, gall-bladder and appendix are more or less equal, should receive the most careful and repeated consideration from the physician, in order that a distinct differential diagnosis may be concluded.

"Fourth—that in the vast majority of instances, a freely movable kidney presents no pathology on the part of the organ itself, and that this immunity may cover a considerable space of time, after there appears symptoms of functional and mechanical disturbances on the part of the stomach, cholecyst and bowel.

"Fifth—That, on account of the wide and uncircumscribed range of pathological symptoms produced by a dislocated kidney, it is more often liable to proceed undiagnosed than all other symptoms producing abdominal lesions.

"Sixth—That permanent and complete relief results only from a correction of the primary lesion with its dependent pathology."

